# From the Role of Microbiota in Gut-Lung Axis to SARS-CoV-2 Pathogenesis

**DOI:** 10.1155/2021/6611222

**Published:** 2021-04-16

**Authors:** Sara Ahmadi Badi, Samira Tarashi, Abolfazl Fateh, Pejman Rohani, Andrea Masotti, Seyed Davar Siadat

**Affiliations:** ^1^Microbiology Research Center, Pasteur Institute of Iran, Tehran, Iran; ^2^Mycobacteriology and Pulmonary Research Department, Pasteur Institute of Iran, Tehran, Iran; ^3^Pediatric Gastroenterology, Hepatology and Nutrition Research Center Research Institute for Children Health, Tehran, Iran; ^4^Children's Hospital Bambino Gesù-IRCCS, Research Laboratories, V.le di San Paolo 15, 00146 Rome, Italy

## Abstract

Severe acute respiratory syndrome-coronavirus 2 (SARS-CoV-2) is responsible for the outbreak of a new viral respiratory infection. It has been demonstrated that the microbiota has a crucial role in establishing immune responses against respiratory infections, which are controlled by a bidirectional cross-talk, known as the “gut-lung axis.” The effects of microbiota on antiviral immune responses, including dendritic cell (DC) function and lymphocyte homing in the gut-lung axis, have been reported in the recent literature. Additionally, the gut microbiota composition affects (and is affected by) the expression of angiotensin-converting enzyme-2 (ACE2), which is the main receptor for SARS-CoV-2 and contributes to regulate inflammation. Several studies demonstrated an altered microbiota composition in patients infected with SARS-CoV-2, compared to healthy individuals. Furthermore, it has been shown that vaccine efficacy against viral respiratory infection is influenced by probiotics pretreatment. Therefore, the importance of the gut microbiota composition in the lung immune system and ACE2 expression could be valuable to provide optimal therapeutic approaches for SARS-CoV-2 and to preserve the symbiotic relationship of the microbiota with the host.

## 1. Introduction

Respiratory tract infections which are responsible for 4 million deaths annually around the world are regarded as health-threatening diseases [1]. The risk of coronavirus respiratory infection has increased following two recent pandemics, which occurred in 2002 by severe acute respiratory syndrome coronavirus-1 (SARS-CoV-1) and in 2012 by the Middle East respiratory syndrome coronavirus (MERS-CoV), with 10% and 37% mortality rates, respectively. In 2019, a new coronavirus infection originating from China (SARS-CoV-2) caused the third coronavirus pandemic [2–4].

It is well established that human health and disease depend on interactions between the immunity system and a coevolved microbial population, called the microbiota. There are symbiotic relationships between the host and the microbiota that colonize the skin and mucosal surfaces, such as gastrointestinal and respiratory tracts [5]. The main commensal microbial community, known as the gut microbiota, is located in the gut, an apparatus with the greatest mucosal surface and interactions with external stimuli. The gut microbiota continuously interacts with the host to preserve homeostasis through the regulation of major signaling pathways (e.g., immune, metabolic, neurologic, and endocrine pathways), as well as by modulating the epigenetic status [6, 7].

The regulating potential of the gut microbiota is not restricted to the gut, but can reach also distant organs, such as brain, liver, kidneys, and lungs. There are various bidirectional pathways, including the gut-brain, gut-liver, gut-kidney, and gut-lung pathways, which give rise to interorgan communication, with microbiota playing a key role [7–10]. In this study, we focused on various aspects of microbiota in the gut-lung axis that can be considered as potential strategies exploitable for preventing, controlling, and treating respiratory infections, especially coronavirus disease 2019 (COVID-19).

## 2. Microbiota Gut-Lung Axis

The gut microbiota can modulate the host physiology, as a function of their composition and derivatives (e.g., metabolites and other components). In a symbiotic state, the gut microbiota reinforces health status through beneficial local and systemic interactions with the host, especially by regulating innate and adaptive immunity and providing critical defense against pathogenic colonization in the gut and other organs (e.g., lungs), thus creating a gut-lung axis [11, 12]. Therefore, disruption of the gut microbiota composition (dysbiosis), which can result from changes in diet or antibiotic consumption, adversely affects the lung microbiota composition, immunity, and pathophysiology and can predispose to respiratory diseases [13]. It has been reported that a lower diversity of gut microbiota composition with significant reduction of short chain fatty acid (SCFA) producer bacteria such as *Faecalibacterium prausnitzii* results to an increased risk of asthma and cystic fibrosis (CF) in children [14, 15]. In this regard, studies showed that lung function and immunity could be influenced by dietary fermentable fiber which affects gut microbiota composition and its metabolome profile especially SCFAs. A high fiber diet clinically reduces mortality by respiratory diseases via modulating inflammatory mediators such as IL-8, [16] IL-6, and C-reactive protein (CRP) [17–19]. Furthermore, it has been reported that fermented milk containing lactic acid bacteria are able to recover natural killer (NK) cell which are reduced for example by smoking [20].

There is an association between early antibiotic administration and development of asthma and allergic diseases caused by an altered microbiota composition [21, 22]. Moreover, the disruption of gut microbiota composition caused by antibiotic consumption reduced pulmonary defense against respiratory pathogens such as *Pseudomonas aeruginosa*, *Staphylococcus aureus*, *Mycobacterium tuberculosis* (*Mtb*), and Influenza A virus through impairment of colonization resistance and lung immunity [23–26].

The respiratory tract is colonized by low-density microbiota, harboring phyla including *Firmicute*, *Bacteroidetes*, *Proteobacteria*, and *Actinobacteria* [27]. Also, *Prevotella*, *Veillonella*, *Streptococcus*, and *Pseudomonas* are dominant genera in healthy lung microbiota [28]. The balance of bacterial immigration and elimination across lungs and their proliferation rate shapes the composition of the lung microbiota [29]. Furthermore, it has been reported that changes in the lung microbiota can influence the gut microbiota via systemic circulation [30].

### 2.1. Role of Microbiota Gut-Lung Axis in Immune System Regulation

**Figure 1:**
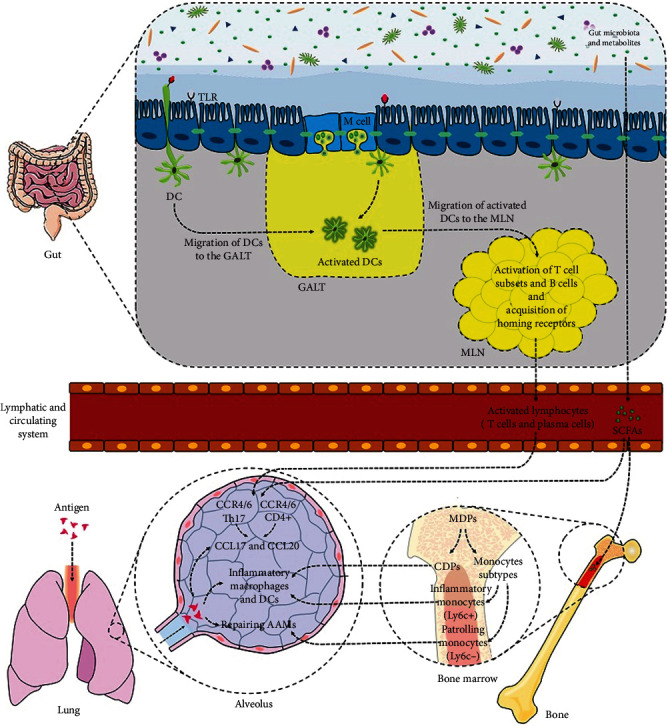
Relationship between the gut microbiota and lung immunity: the interaction between the intestinal commensal bacteria and establishing of lung immunity is mediated by various factors, including PAMPs, PRRs, SCFAs, intestinal integrity, and immune cells of the lamina propria. In a normal state, DCs are continuously sampled from the lumen through M-cell activity, extension of dendrites, and the gut barrier function, which determine bacterial/PAMP translocation. After DC sampling, these cells migrate to GALT and then MLN to regulate differentiation and homing of lymphocytes (T and B cells) depending on the released certain cytokines in respect to gut microbiota composition. The activated T and B cells are distributed in the lungs via circulation. Also, the levels of CCL20 and CCL17, which are produced by the lungs after microbial exposure, contribute to imprinting of T cell subsets, based on the cognate CCRs. Furthermore, SCFAs can penetrate into the bone marrow and influence lung immunity by affecting MDP differentiation to inflammatory or anti-inflammatory immune cells. Inflammatory macrophages and DCs in the lungs are derived from CDPs and Ly6C^+^ inflammatory monocytes. Alternatively, activated macrophages (AAMs) are anti-inflammatory immune lung cells, derived from Ly6C^−^ patrolling monocytes subtypes.

The gut microbiota has local and systemic regulatory effects on innate and adaptive immune systems due to the presence of almost 70% of immune cells in the gastrointestinal tract, especially in the *lamina propria* [12]. This interaction is governed by the gut barrier function, which involves the epithelium layer, mucous, and immunomodulatory mediators. The epithelium layer consists of specialized intestinal epithelial cells with different functions such as absorptive enterocytes (for nutrient absorption and maintenance of epithelial integrity), Paneth cells (for secretion of antimicrobial peptide such as *α*-defensin), goblet cells (for secretion of mucin and trefoil peptides), microfold cells (for secretion of Ig-A and presenting antigens to dendritic cell (DC)), and enteroendocrine cells (for production of hormones such as glucagon-like peptide 1 (GLP-1) and GLP-2). These cells are connected together by tight junctions (Tj) proteins (e.g., occludin, zonula occludens (ZOs), and junctional adhesion molecules (JAM)) to create a dynamic and complex intestinal interface (gut barrier) for the regulation of microbiota-host interaction. Dysregulation of Tj protein expression and localization, mucin thickness, proliferation, and renewal of epithelial lining can lead to an increase in the gut barrier permeability [31]. Gut microbiota composition, metabolites, and immunogenic components, known as microbe-associated molecular patterns (MAMPs), control both the gut barrier function and inflammatory status (Figure 1) [32]. Furthermore, gut barrier function and immune system are considered two important factors to shape microbiota composition [33].

Therefore, dysbiosis disrupts the gut barrier function and induces hyperpermeability of the epithelium lining (which is also considered as a dysbiosis inducer), leading to the increased activation of innate immune system receptors, called pattern recognition receptors (PRRs), which are expressed by immune and nonimmune cells. It also favors Th and Th17 cell differentiation over regulatory T cell (Treg) differentiation by DC sampling from the lumen into the lamina propria [34]. The elevated proinflammatory responses, resulting from this event, are considered as the starting point of various inter- and extraintestinal disorders induction and development [7, 32] (Figure 1). Moreover, the regulatory effect of the gut microbiota on the extraintestinal T cell population, which contributes to systemic immunity control, has been reported. Several studies on animal models have shown that a specific strain of the gut microbiota affects differentiation of T cell subsets. For example, expansion of CD4^+^ T cells, Treg cells, and Th17 cells has been attributed to the colonization of *Bacteroides fragilis*, Clostridia, and segmented filamentous bacteria (SFB) in germ-free mice, respectively [35–37].

Tissue-specific homing of lymphocytes, mediated by chemokines and cognate receptors, can be determined by DC function, which is dependent on the gut microbiota composition. DCs are continuously sampled from the microbiota and pathogen-derived MAMPs. They migrate to draining lymph nodes, where they induce T cell activation and differentiation (Figure 1) [38]. Chemokine (C-C motif) ligand 20 (CCL20) is expressed by various tissues (e.g., epithelial cells of the gut and lungs at the basal level) and increased by toll-like receptor (TLR) activation and proinflammatory signals. The activation of CCR6 by CCL20 induces homing of CD4^+^ T cells and DCs in the gut-lung axis [39]. Evidence shows that lung DCs are involved in imprinting of CCR4 T cells by increasing the level of CCL17, a cognate ligand produced by the lungs and increased after infection (Figure 1) [40].

The gut microbiota plays a determinative role in the regulation of IgA-producing plasma cells from activated and differentiated B cells, which specifically release antibodies and shape the mucosal immunity. Sensitized B cells which are located in lamina propria (Peyer's patches) by inhaled and gut microbiota antigens can reach the respiratory tract to produce specific IgAs and translocate onto the mucosal surface. As mentioned earlier, the gut microbiota composition is a key factor in the gut barrier function, regulating interactions between antigens and the immune system [41, 42]. Therefore, the conserved symbiotic relationship of the gut microbiota with immunity reinforces lung immunity immune system against respiratory bacterial and viral pathogens.

The intact bacteria and immunogenic components can diffuse through the intestinal lymphatic system toward systemic circulation and the lungs. Therefore, bacterial clearance of alveolar macrophages, neutrophil recruitment, and antibacterial factors, derived from the bronchus epithelium, depend on the gut microbiota composition and its derivatives [43]. Moreover, the interplay between diet and the gut microbiota determines immunity, as reflected in the lung physiology [44]. Generally, diet is a key factor in the gut microbiota composition and SCFA profile. For example, saturation of fatty acids in the diet can affect luminal immunity, since saturated and unsaturated fatty acids are considered as TLR agonists and antagonists, respectively, and have an impact on the gut microbiota composition [45].

Moreover, SCFAs which are mainly produced from dietary fibers by bacterial fermentation, are multitasking molecules, associated with the maintenance of immune homeostasis through various mechanisms: (i) reinforcement of the intestinal epithelium integrity; (ii) increasing the level of mucin-producing goblet cells; (iii) elevating the intestinal IgA production; (iv) improvement of intestinal cell survival and repair via NLRP3 inflammasome activation; (v) activation of macrophage and DC signaling by G-protein coupled receptors (GPR109A) for interleukin-10 (IL-10) production; and (vi) induction of intestinal Foxp3 Treg cell differentiation by GPR43 sensing [46–49].

The epigenetic role of SCAFs (butyrate) in regulation of intestinal inflammation has been reported to induce the suppression of histone deacetylase (HDAC) activity, followed by the induction of colonic Foxp3^+^ Treg cell expression [50]. Moreover, in a symbiotic state, SCFAs preserve the desired intestinal bacterial community through intestinal hypoxia, resulting from dominancy of colonocyte metabolism by fatty acid beta-oxidation and oxidative phosphorylation in the mitochondria [51, 52]. Furthermore, the gut microbiota and its SCFAs have hematopoiesis-regulating effects in the bone marrow. Circulating SCFAs can penetrate into the bone marrow and affect lung immunity in allergic airway diseases and respiratory infections (e.g., influenza virus infection) through differentiation of common DC precursors (CDPs), macrophages, and DC progenitors (MDPs) [53]. In the bone marrow, CDPs and two monocyte subtypes, including Ly6C^+^ (Gr1^+^) inflammatory monocytes and Ly6C^−^ (Gr1^−^) patrolling monocytes, are derived from MDPs. In inflammatory conditions, such as viral infections, severe tissue damage is induced by uncontrolled immune responses, such as increased differentiation of inflammatory Ly6C^+^ monocytes to inflammatory DCs and macrophages, which can trigger the immunopathology of the lungs [54].

Trompette and collaborators studied the effect of gut microbiota on bone marrow hematopoiesis and effective lung immunity and found that high-fiber diets and SCFA metabolites affect the bone marrow hematopoiesis by increasing the level of Ly6C^−^ (Gr1^−^) patrolling monocyte subtypes. The elevated level of patrolling monocytes dampens tissue damage by increasing the airway count of alternatively activated macrophages (AAMs), which participate in tissue protection and repair (Figure 1). Moreover, SCFAs enhance the function of CD8^+^ effector T cells against influenza infection by altering T cell metabolism [55].

Desaminotyrosine (DAT) is another microbial metabolite, produced by flavonoid and amino acid metabolism. This metabolite is correlated with type-I IFN activity, which plays a key role in viral immunity. *Clostridium orbiscindens*, a member of the gut microbiota, can produce DAT from flavonoids and has a protective effect against influenza infection and decreased mortality in influenza-infected mice [56]. Besides the gut microbiota metabolites, studies on extracellular vesicles (EVs), which can be derived from the intestinal gut microbiota as new systemic mediators, are notably growing. Generally, EVs are nanosized particles, containing enclosed MAMPs, hydrolytic enzymes, and nucleic acids, which can diffuse across the body to regulate the host function, especially immune responses [57].

Many reports indicate that EVs derived from important gut microbiota members, including *Bacteroides fragilis*, *Akkermansia muciniphila*, and *Faecalibacterium prausnitzii*, may have important immunomodulatory effects [58–60]. Therefore, the assessment of the gut microbiota EV patterns can be potentially used for screening disease progression [61, 62]. Overall, the composition of the gut microbiota, associated with diet, can determine lung immunity by changing innate and adaptive immune responses. Therefore, there are prominent aspects of microbiota gut-lung axis that can be considered as promising targets in the prevention, control, and treatment of SARS-CoV-2 infection.

## 3. Respiratory Diseases Controlled by Microbiota Gut-Lung Axis

There are various reports discussing the changes of gut and lung microbiota during respiratory diseases (caused by bacterial and viral pathogens). In this regard, Dumas et al. studied the importance of microbiota in acute lung infections, such as pneumonia, induced by *Pseudomonas aeruginosa*, *Streptococcus pneumoniae*, and *Klebsiella pneumoniae* in antibiotic-treated germ-free mice [43]. The protective activity of the gut and lung microbiota against pneumonia is mainly mediated by nucleotide-binding oligomerization domain-like receptor and IL-17A-driven granulocyte macrophage-colony-stimulating factor (GM-CSF) signaling pathways, which promote innate immune responses, especially pathogen clearance by alveolar macrophages [63].

In addition to respiratory bacterial pathogens, viral infections, caused by influenza virus, respiratory syncytial virus (RSV), SARS-CoV-1, MERS, and SARS-CoV-2, which may be followed by secondary bacterial pneumonia, are important causes of morbidity and mortality worldwide. Overall, the interactions between respiratory pathogens, microbiota, and immune system can determine the severity of respiratory diseases, a relationship that is also closely linked to antibacterial and antiviral immune responses (e.g., type I IFN, type II IFN, and IL-17), antibody responses, and colonization resistance by the gut and lung microbiota.

Microbiota is among the major determinants of lung immunity, and respiratory viral infections can affect the gut and lung microbiota composition. Influenza-infected mice exhibited an altered intestinal microbiota composition as a function of the increased abundance of *Enterobacteriaceae* and decreased amount of SFB. These induce intestinal immune injury due to the involvement of the CCL25-CCR9 axis in recruiting lymphocytes (i.e., lung-derived CD4^+^ effector T cells secreting IFN-*γ*) into the intestine and Th17 cells promotion [64].

Ichinohe and collaborators emphasized the role of gut microbiota in the regulation of antiviral responses of CD4, CD8, and B cells against respiratory influenza virus infection, especially through inflammasome activation by providing proper MAMPs for prime immunity. They found that the antibiotic-induced microbiota changes resulted in failure in the production of inflammasome-dependent cytokines. These changes also impaired homeostasis and migration of lung DCs into lymph nodes to prime T cell responses against influenza virus in mice [26]. In this regard, Wang and coworkers studied the possible protective role of the lung microbiota in subsequent lung injury and lethal inflammation, resulting from influenza infection. They reported a significant decrease in lung injury caused by *Staphylococcus aureus*, a common colonizer of the upper respiratory tract, by promoting M2 polarization of alveolar macrophages, followed by anti-inflammatory cytokines [65].

The impact of viral pulmonary infections on the gut microbiota composition has been attributed to changes in systemic immune signals and bacterial translocation to the gut [66]. Therefore, modulation of the gut microbiota composition, based on the pre/probiotic interventions, has therapeutic effects on respiratory viral infections such as pneumonia. Furthermore, the potential of postbiotics intervention in the modulation of immunity in various diseases including asthma, COPD, and respiratory infections has been reported. Postbiotics are defined as microbial components, soluble factors, and metabolites which are, respectively, secreted or released by live microbial cell or its lysate and inactivated [67, 68]. There are several reports demonstrating the beneficial effects of probiotics in influenza-infected mice. A previous study showed that intranasal or oral administration of *Lactobacillus plantarum* DK119 conferred protective defense against a lethal dose of influenza A virus by modulating DC and macrophage activities and also increasing the levels of IL-12 and IFN-*γ* in the bronchoalveolar fluid [69].

Moreover, oral administration of *L*. *paracasei* CNCM I-1518 strain preactivates the immune system to clear more rapidly the influenza virus by early stimulation of proinflammatory cytokines and recruitment of immune cells. In a previous study, after viral infection *L*. *paracasei* provided better tissue homeostasis through IL-13 and IL-15 production by T cells, which promoted hyperplasia of lung epithelial cells during inflammation, compared to control-fed mice [70]. Smith and collaborators showed higher mortality rate and decreased antiviral responses against influenza infection in high-fat-diet-induced obese (DIO) mice, compared to the lean group [71]. Additionally, Yoda et al. targeted the gut microbiota by oral administration of heat-inactivated *L. gasseri* TMC0356 (postbiotic) to alleviate obesity-induced lung immune disruption in DIO mice [72]. These findings emphasize the increased susceptibility of obese mice to respiratory viral infections due to immune dysregulation controlled by the gut microbiota.

## 4. Effects of Microbiota on Vaccination for Viral Respiratory Infections

Vaccine efficacy is determined by various factors, including genetic background, lifestyle, mode of delivery, nutrition, age, gender, geographical region, and economic status, which play critical roles in the composition of gut microbiota. As mentioned earlier, innate and adaptive immune responses are controlled by gut microbiota, which has immunomodulatory effects. Evidence shows that differences in vaccine efficacy between certain populations with distinct characteristics affect the gut microbiota and immune status. In this regard, previous studies have reported differences in the rotavirus vaccine efficacy between countries [73, 74].

The beneficial effects of pre- and probiotic interventions have been reported to increase immune responsiveness to respiratory viral vaccination, as shown by the improvement of innate and adaptive immune responses in animal models and clinical trials [75, 76]. Moreover, previous studies have reported the orchestrating role of the gut microbiota in TLR5 activation, plasma cell differentiation, and antibody responses to influenza virus, which are defective in antibiotic-treated Trl5(-/-) mice and are improved by oral administration of flagellated *E. coli* [77]. Moreover, recombinant probiotic strains have been introduced as adjuvants for edible vaccines to provide safer and better immunization [78, 79]. Lei et al. designed a recombinant *Lactococcus lactis* strain, expressing H5N1 hemagglutinin antigen, as a stable oral vector of influenza vaccine. They identified higher levels of hemagglutinin-specific IgA antibodies in the serum and fecal samples of mice [80].

There are several clinical trials confirming the immunomodulatory role of probiotic pretreatments in increasing immune responses to influenza vaccination. For example, pretreatment with *Lactobacillus rhamnosus* GG, *Bifidobacterium animalis*, and *Lactobacillus paracasei* before influenza vaccination improved the vaccine immunogenicity against the H3N2 influenza strain [81, 82]. Nasal microbiota participates to determine immunogenicity of vaccines. It has been shown that administration of the Live, Attenuated Influenza Vaccine (LAIV) induces changes in the upper respiratory microbiota, producing specific influenza antibodies. Salk et al. demonstrated a significant association between the increased alpha diversity and the presence of *Lactobacillus helveticus*, *Prevotella melaninogenica*, *Streptococcus infantis*, *Veillonella dispar*, and *Bacteroides ovatus* in the nasal microbiota and specific IgGs after LAIV administration [83].

## 5. SARS-CoV Immunopathology and Microbiota

Coronaviruses (CoV) contain a positive-sense single-stranded RNA genome, which is enclosed within an envelope, containing spike glycoprotein (S), membrane protein (M), envelope protein (E), and in some cases, hemagglutinin-esterase (HE). These viruses are divided into four subgroups of *α*, *β*, ɣ, and *δ*, based on the genotypes and serological properties, with *α* and *β* subtypes causing human infections [84]. In the past decade, the world has experienced three life-threatening CoV infections, caused by SARS-CoV-1, MERS-CoV, and a novel betacoronavirus, called SARS-CoV-2, which has caused significant mortality during the current pandemic.

The genome sequencing of SARS-CoV-2 revealed 79.5% similarity to the SARS-CoV genome [85]. The entry of SARS-CoV into the host cells is mediated by binding of variable receptor-binding domain (RBD) of S protein to Angiotensin-converting enzyme 2 (ACE2) receptor, which is expressed in the heart, lungs, kidneys, and gastrointestinal tract [86]. In SARS-CoV-2 infection, inflammatory responses begin in type II lung pneumocytes after the virus binds to ACE2. The proteolytic activity of type 2 transmembrane protease (TMPRSS2) requires viral entry through ACE2 cleavage and S protein [87].

**Figure 2:**
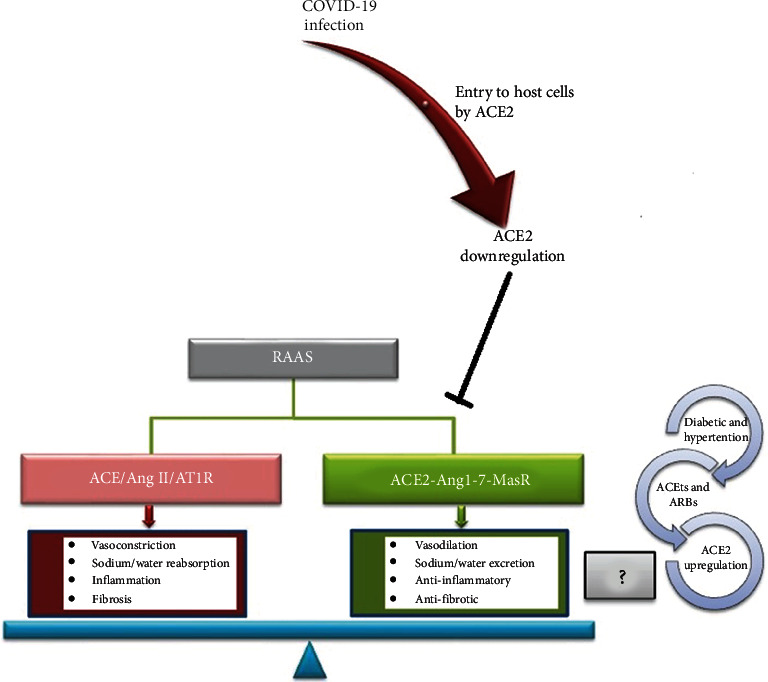
The function of two arms of renin-angiotensin-aldosterone system (RAAS) axis: the RAAS consists of two pathways including (i) ACE/Ang II/AT1R: in the pathway, Ang II which cleaved from Ang I by ACE activity, interacts with AT1R to induce vasoconstriction, inflammation, and fibrosis. (ii) ACE2-Ang1-7-MasR: in the pathway Ang 1-7 negatively regulate RAAS through promotion of vasodilation, anti-inflammatory and antifibrotic effects by interaction with MasR. Ang 1-7 are produced from cleavage of Ang II by ACE2 or metabolized of inactivated Ang 1-9 (cleaved from Ang I by ACE2) by ACE. The balance between two arms determines healthy state. In COVID-19 infection, ACE2, main receptor to SARS-CoV-2 entrance, is significantly decreased which results to inhibition of protective function of ACE2-Ang1-7-MasR arm. In opposite, the increased level of ACE2 resulted from ACEI and ARB medication in diabetic and hypertensive patients is considered as a double-edged sword which has been raised a big question: which aspects of increased ACE2 could be dominated during COVID-19 infection? increased susceptibility to viral infection or protective potential in RAAS system.

ACE2 has a protective and regulatory role in Renin-angiotensin-aldosterone system (RAAS) mainly through two pathways: (i) Angiotensin-converting enzyme (ACE) cleaves angiotensin I (Ang I) into Ang II that interacts with Ang II type 1 receptor (AT_1_R). The activation of this way (ACE/Ang II/AT1R) leads to higher blood pressure and inflammation caused by increased vasoconstriction, renal reabsorption of sodium/water and induction of proinflammatory chemokines [88]. (ii) ACE2-Ang1-7-MasR pathway where ACE2 is a key enzyme converting Ang II into Ang 1-7 peptide, whereas Ang I is converted into inactive Ang 1-9. After this step, Ang 1-9 are metabolized to Ang1-7 by ACE. The peptide is recognized by Mas receptor to negatively regulate RAAS system in many lung and heart functions and blood pressure homeostasis. ACE2-Ang1-7-Mas pathway exerts the beneficial effect against hypertension and acute lung injury by inactivation Ang II which is upregulated in these conditions (Figure 2) [89, 90]. It has been shown that SARS-CoV infection significantly downregulates ACE2 in the lungs [91]. In this state, also, the production of Ang1-7 which is regulated by ACE2 activity is diminished. Therefore, the lack of ACE2-Ang1-7-Mas pathway activity leads to loss of its protective effects, and ACE/Ang II/AT1R pathway is overactivated and accumulates Ang II. These cascade events are observable during pulmonary and acute lung injury and fibrosis [92, 93].

Evidence shows that hypertension and diabetic patients, who therapeutically receive ACE inhibitors (ACEIs) and AT_1_R blockers (ARBs), have elevated ACE2 levels and could be at high-risk for COVID-19 infection. In these patients, Ang II which causes hypertension and inflammation is increased, and ACE/Ang II/AT1R is activated. ACE2 controls Ang II level and activity and balance RASS by cleavage Ang II to Ang 1-7 peptides to exert protective effect by interaction with MasR [94]. It has been shown that ACE2 is insensitive to inhibition by ACEIs [95]. Moreover, ACEI medication and ARB medication increase ACE2 gene expression and activity which led to an overactivation of ACE/Ang II/AT1R by inactivation of Ang II. Upregulation of ACE2 in these patients can facilitate the SARS-CoV-2 entry [96, 97]. As mentioned before, SARS-CoV-2 significantly decreases ACE2 after entry into the lung and attenuates its protective effect against lung injury and failure. ACE2 could act as a double-edged sword for these patients due to its dual function as a gate of SARS-CoV-2 entry and also protecting of lung injury and cardiovascular and renal complication in diabetic patients [98]. Nevertheless, European Medicines Agency (EMA) suggested to maintain these medications in diabetic and hypertensive patients due to the increased mortality resulting from the withdraw of these medications [99] (Figure 2). A big question arises: can the increase of ACE2 levels after ACEIs and ARBs medication have adverse (by facilitation of SARS-CoV-2 entry) or beneficial (by protective role in RAAS system) effect in diabetic and hypertensive patients during COVID-19 infection?

Also, ACE2 plays a key role in gastrointestinal inflammation and the gut microbiota composition [100]. A recent study highlighted the critical role of the gut microbiota in the colonic ACE2 gene expression in gnotobiotic rats and reported its implication on the COVID-19 pathology through the gut-lung axis [101]. The abundance of *Bacteroides* showed a negative correlation with the COVID-19 severity and the fecal load of SARS-CoV-2 [102]. *Bacteroides* species including *B. dorei*, *B. thetaiotaomicron*, *B. massiliensis*, and *B. ovatus* are able to downregulate ACE2 expression in the colonocytes of mice [103]. These findings suggest the possible protective role of the *Bacteroides* spp. as important gut microbiota member against COVID-19 infection by downregulation of ACE2 and reduction of SARS-CoV-2 entry [92]. In fact, since SARS-CoV-2 entry is linked to ACE2 expression level, an increased level may promote the viral entry, whereas its downregulation reduces the ACE2-Ang1-7-Mas pathway and further protect from lung injuries during SARS-CoV-2 infection.

The pathogen-associated molecular patterns (PAMPs) of viral infection are recognized by two innate immune receptors (PRRs), including TLR3 and retinoic acid-inducible gene-like-I- (RIG-I-) like receptors (RLRs), which sense viral RNA to induce type I IFN (IFN-*α* and IFN-*β*), as a major antiviral and immunomodulatory mediator, promoting macrophage, NK cell, B cell, and T cell activities [104]. It has been shown that TLR3 activates IRF3 and NF-*κ*B to express type I IFN and trigger proinflammatory responses through the TRIF-dependent pathway as adaptor protein [105]. TLR4 activation in the MyD88-dependent TRIF signaling pathway occurs during respiratory viral infections.

TLR4 expression increases after an immune response to a viral infection in bronchial epithelial and alveolar cells [106]. TLR signaling pathways promote the production of IFN-*α*, IFN-*β*, IL-6, TNF, IFN-*γ*, CCL5, and IFN-stimulated genes, which are produced during acute respiratory distress syndrome (ARDS) and viral infections [107]. In this regard, Totura et al. reported that the absence of TRIF- or MyD88-dependent TLR pathways resulted in the death of mice infected with SARS-CoV. They suggested that balance between the two arms of TLR signaling provides effective antiviral responses to severe SARS-CoV. Their results demonstrated the possible protective effect of TLR3 and TLR4 agonists as protective therapeutic strategy against SARS-CoV infection [108].

RLRs, such as RIG-I and melanoma differentiation-associated protein 5 (MDA5), are cytosolic PRRs recognizing viral dsRNA. They contain C-terminal domains (CTD) and N-terminal caspase recruiting domains (CARD) to sense RNA and activate mitochondrial antiviral signaling proteins (MAVS) as downstream adaptor proteins, promoting antiviral responses (e.g., type I IFN) [109]. In this regard, Lu highlighted the potential of SARS-CoV in inhibiting IFN responses by N protein, which contributes to SARS-CoV pathogenesis [107].

DCs play key roles in combining innate and adaptive immune responses by affecting T cell and B cell activation. Generally, the polarization of DCs affects the outcomes of viral infection. The conventional DCs (cDCs) and plasmacytoid DCs (pDCs) induce the production of type I IFN by PRR recognition of viral PAMPs, including TLR3, RIGI, MDA5, and TLR7-9 [110]. In a normal state, there are three DC subtypes in the lungs, including CD103^+^ cDCs, CD11b^+^ cDCs, and pDCs, while in inflammatory conditions, monocyte-derived DCs (moDCs) are recruited into the lungs. The subtypes of DCs and polarization of T cells are determined by the type of respiratory virus and DC expression of PRRs. Differentiation of T cells into CD4^+^ T, CD8^+^ T, Treg, and Th17 cells is controlled by DC function through activation of DC PRRs and cytokine and chemokine receptors [111]. Therefore, skewing of T cell polarization can result in host damage and increase the severity of disease during viral respiratory infections.

CD8^+^ T cells, which are essential in clearing virus-infected lung cells and promote immune injury, are near to 80% of infiltrated immune cells into the lungs in SARS-CoV patients [112]. The production of SARS-CoV is activated in B cells through CD4^+^ T cell function. In this regard, Chen et al. reported the significance of CD4^+^ T cells in mice infected with SARS-CoV, as depletion of these cells resulted in increased pneumonia and delayed respiratory viral clearance, associated with decreased neutralizing antibodies and recruitment of immune cells to the lungs [113]. Also, SARS-CoV-specific CD4 and CD8 memory T cells may play a vital role in protecting against reinfection with SARS-CoV. Channappanavar et al. found that unlike CD4^+^ T cells, CD8^+^ memory T cells remain up to six years after SARS-CoV infection, mediating protective effects against lethal SARS-CoV infection [114].

Considering the importance of microbiota in the gut-lung axis in COVID-19 patients, reports about this topic have progressively increased. The metatranscriptome sequencing of bronchoalveolar lavage fluid showed similar microbiota between COVID-19 and community-acquired pneumonia (CAP) patients. The dominance of pathogens or higher count of oral and upper respiratory tract symbiotic bacteria was reported in these patients, compared to the healthy controls [115]. Although the main target of SARS-CoV-2 is the lung, some meta-analyses have reported gastrointestinal manifestations and the presence of SARS-CoV-2 RNAs in anal swabs and stool samples of COVID-19 patients [116, 117].

Several studies demonstrated the presence of a gut microbiota alteration in stool samples of COVID-19 patients, compared to healthy individuals. In this regard, Zou et al. reported that the gut microbiota of COVID-19 patients contained less beneficial commensal bacteria, such as *F. prausnitzii*, and was enriched with bacteremia-associated pathogens, which could increase the severity of disease course due to secondary bacterial infections [102]. Also, in a pilot study, it has been documented a higher abundance of *Parabacteroides merdae*, *Bacteroides stercoris*, *Alistipes onderdonkii*, and *Lachnospiraceae*, SCFAs producer bacteria, in fecal samples of SARS-CoV-2 patients with low to none infectivity [118]. According to a previous study, a significant diversity reduction was observed in the gut microbiota of COVID-19 patients (similar to patients with H1N1 infection), compared to healthy subjects. Interestingly, gut microbiota signature was significantly different between patients with SARS-CoV-2, patients with H1N1 infection, and control individuals, as a function of the abundance of opportunistic pathogens [119]. It is also important to avoid unnecessary antibiotic administrations that may cause a potential reduction of symbionts and determine a gut microbiota dysbiosis during COVID-19 treatment [102, 120]. Furthermore, a healthy diet rich in fibers (whole grains and vegetables) should be considered as beneficial for COVID-19 patients' treatment due to their significant anti-inflammatory potential and ability to target the microbiota-lung axis [121]. Since gut microbiota finely tune local and systemic immune responses and alter its composition, it may have an important role in the host sensitivity toward COVID-19, secondary bacterial infections, and organ failure and damage.

## 6. Conclusions

The crucial role of gut microbiota in establishment and providing innate and adaptive immunity in the respiratory tract has been demonstrated. According to the composition of the gut microbiota and its products (e.g., metabolites and components), pulmonary immune responses can be explained through various pathways: (i) ACE2 expression; (ii) activation of PRRs, such as TLRs, NLRs, and RLRs for producing antiviral responses, such as type I IFN and proinflammatory cytokines; (iii) translocation of CDP and MDP subtypes from the bone marrow to the lungs; and (iv) activation and homing of T and B cells from the gut-associated lymphoid tissue to the lungs. Moreover, the presence of commensal bacteria in the respiratory tract may affect alveolar macrophage polarization (M2) to dampen lung injury, induced by elevated inflammatory responses. On the other hand, the beneficial role of probiotic pretreatment in vaccination efficacy against viral respiratory tract infections has been discussed. Therefore, differences between populations (reflecting the microbiota composition) could be attributed to the sensitivity and severity of SARS-CoV-2 infection. Finally, further research on the study of microbiota gut-lung axis is essential to design a therapeutic strategy and develop a vaccine against SARS-CoV-2 infection.

## Data Availability

The data used to support the findings of this study are available from the corresponding authors upon request.
